# Evaluation of Gelatin as a Biostimulant Seed Treatment to Improve Plant Performance

**DOI:** 10.3389/fpls.2018.01006

**Published:** 2018-07-27

**Authors:** Hiromi T. Wilson, Masoume Amirkhani, Alan G. Taylor

**Affiliations:** Section of Horticulture, School of Integrative Plant Science, New York State Agricultural Experiment Station, Cornell AgriTech, Cornell University, Geneva, NY, United States

**Keywords:** amino acid, biostimulant, gelatin, hydrolyzed collagen, nitrogen uptake, seed enhancement, vegetable crops

## Abstract

The effect of gelatin, used as a biostimulant, was investigated on plant growth in greenhouse studies. Biostimulants are materials that stimulate plant growth, and gelatin, an animal protein hydrolysate, is classified as one type of biostimulant. Gelatin has a unique amino acid composition with a high percentage of proline and hydroxyproline. In a series of experiments gelatin capsules (#3 hard gelatin) containing 7.1 mg nitrogen each, were placed adjacent to seeds of different crop species, at sowing time in individual growing containers and several growth parameters were measured. Different types of hydrolyzed collagen, including granulated gelatin, gelatin hydrolysate, and amino acid mixtures simulating the composition of gelatin were compared on cucumber plant growth. In addition, amino acid mixtures without proline, hydroxyproline, or applied in combination were investigated on cucumber growth. All capsule treatments significantly enhanced crop growth compared to the non-treated control. The treatment with two gelatin capsules placed adjacent to each seed increased shoot dry weight of cucumber, pepper, broccoli, tomato, arugula, and field corn, by 138, 244, 50, 45, 41, and 18 percent, respectively. In an experiment with cucumber alone, there was a positive linear relationship between the number of gelatin capsules from 0 to 3 capsules on plant growth and plant nitrogen content. Cucumber growth and plant nitrogen content was greater from the hydrolyzed collagen treatment compared with the low molecular weight gelatin hydrolysate, a mixture of amino acids or urea and all treatments provided an equivalent amount of nitrogen. Proline and/or hydroxyproline were not responsible for the biostimulant effect. In summary, gelatin provided nitrogen that enhanced plant growth. Moreover, gelatin was an effective biostimulant as the plant growth and nitrogen content was greater from two gelatin capsules compared to amino acid mixture of the same proportion and amount as the gelatin.

## Introduction

Seed enhancement is a term widely used in the industry to describe beneficial techniques performed to seeds post-harvest, but prior to sowing (Taylor et al., 1998). Seed enhancements include plant biostimulants, a broad class of substances and microorganisms that enhance plant growth. The European Biostimulants Industry Council (EBIC) developed a definition for plant biostimulants; “substance(s) and/or microorganisms whose function when applied to plants or the rhizosphere is to stimulate natural processes to enhance/benefit nutrient uptake, nutrient efficiency, tolerance to abiotic stress, and crop quality. Biostimulants have no direct action against pests, and therefore do not fall within the regulatory framework of pesticides" (du Jardim, 2012; European Biostimulants Industry Council [EBIC], 2014). Gelatin, an animal based protein hydrolysate (hydrolyzed collagen) is one category of plant biostimulants.

Gelatin is defined as a mixture of peptides and proteins that are generally derived from partial hydrolysis of collagen obtained from connective tissues of animals which can include skin and bones (Gelatin Manufacturers Institute of America, 2012). Gelatin is soluble in water and in most polar solvents. A measure of gelatin strength is termed ‘Bloom.’ The higher the Bloom number, the stronger the gel. The Bloom number is positively related to average molecular mass (Ward and Courts, 1977; Gelatin Manufacturers Institute of America, 2012). The amino acid contents of protein hydrolysates vary depending on the production method as well as the source of the material. Animal based hydrolysates, such as gelatin, contain a high proportion of proline and glycine, while plant based hydrolysates contain a high proportion of glutamine and arginine (Parrado et al., 2008; Ertani et al., 2013a; Calvo et al., 2014). Gelatin contains 18 amino acids (**Table 1**) and the molecular size distribution for amino acids present in hydrolyzed gelatin ranges from 89.1 to 204.2 g mol^−1^ (Schrieber and Gareis, 2007). About 50% of the total amino acids in gelatin are glycine, proline, and hydroxyproline (**Table 1**). A gelatin molecule is composed of an average of 1,000 amino acids per chain and there are a few disulfide bonds in gelatin molecular structure (Belitz et al., 2004). Hydroxyproline is especially important for its primary role in the structure and maintenance of gelatin. Hydroxyproline is found in the cell wall proteins in plants (Lamport, 1965), and is derived from glycoprotein present in the primary cell walls (Kieliszewski and Shpak, 2001).

**Table 1:** List of amino acids and their relative amount in 1 g of gelatin protein (Gelatin Manufacturers Institute of America, 2012).

Amino acid	Amino acid (mg/g) of gelatin protein	Amino acid equivalent of one capsule (mg)
Alanine	113	45
Arginine	90	3.6
Aspartic acid	67	2.7
Glutamic acid	116	4.6
Glycine	272	10.8
Histidine	7	0.3
Hydroxylysine	8	0.3
Hydroxyproline	133	5.3
Isoleucine	16	0.6
Leucine	35	1.4
Lysine	44	1.8
Methionine	6	0.2
Phenylalanine	25	1.0
Proline	155	6.1
Serine	37	1.5
Threonine	24	0.8
Tyrosine	2	0.1
Valine	28	1.1

*The calculated amino acid content of one gelatin capsule considering the moisture content of a gelatin capsule at 14% (Buice et al., 1995). Weight of one gelatin capsule is 45 mg, with 7.1 mg nitrogen content and 17.6 mm × 5.5 mm dimension.*

Protein hydrolysate and other protein-based product applications were reported to enhance plant growth and yield in field tomato (Parrado et al., 2008), greenhouse tomato (Koukounararas et al., 2013), papaya (Morales-Payan and Stall, 2003), maize seedlings (Ertani et al., 2013a,b), broccoli, and tomato seedlings (Amirkhani et al., 2016, 2017), and hydroponic lettuce (Colla et al., 2013). Protein hydrolysates were also reported to have ameliorating effects on abiotic stress in plants (Ertani et al., 2013a). An alfalfa plant-derived biostimulant increased maize plant biomass under salinity stress, and enhanced K^+^ accumulation and reduced Na^+^ accumulation in roots and leaves (Ertani et al., 2013b). Combined application of plant-derived protein hydrolysate and beneficial microorganisms improved lettuce root system architecture, chlorophyll synthesis and proline accumulation and enhanced lettuce tolerance to salinity and alkalinity (Rouphael et al., 2017). Perennial ryegrass treated with Macro-Sorb Foliar (FOLIAR^TM^), an animal membrane hydrolysate, showed membrane stability and increased photosynthetic capacity when subjected to high temperature stress (Kauffman et al., 2007).

Hard gelatin capsules, routinely used in the pharmaceutical industry for medications were developed as a novel approach to deliver single or multiple seeds for greenhouse production (Trias and Takahashi, 2014). Sowing multiple seeds in a single dispersal unit in the same growing container has been used to facilitate sowing different varieties of the same crop. Trias and Takahashi (2014) reported enhanced plant growth from seeds sown in gelatin capsules compared to control seeds. However, in preliminary greenhouse studies, seeds placed inside gelatin capsules initially decreased the germination and seedling emergence rate, followed by enhanced growth (Wilson and Taylor, unpublished). The objective of this study was to investigate the biostimulant effect of the gelatin chemistry on plant growth without the deleterious effect of seed encapsulation. Therefore, capsules were placed adjacent to a seed in all experiments. In addition, only single seeds were placed near each gelatin capsule. The focus of this study was to first characterize the influence of gelatin capsule treatments on plant growth measured as dry weight and leaf area of several crops. Different types of hydrolyzed collagen [with molecular weight distribution (MWD) characterized] amino acids and nitrogen fertilizer were also evaluated as biostimulants with respect to plant dry weight and nitrogen content in cucumber. In these studies, gelatin was investigated both as a source of nitrogen and as a biostimulant enhancing plant nitrogen uptake.

## Materials and Methods

### Experiment 1: Capsule Treatments and Above Ground Growth of Selected Vegetable Crops and Field Corn

**FIGURE 1:**
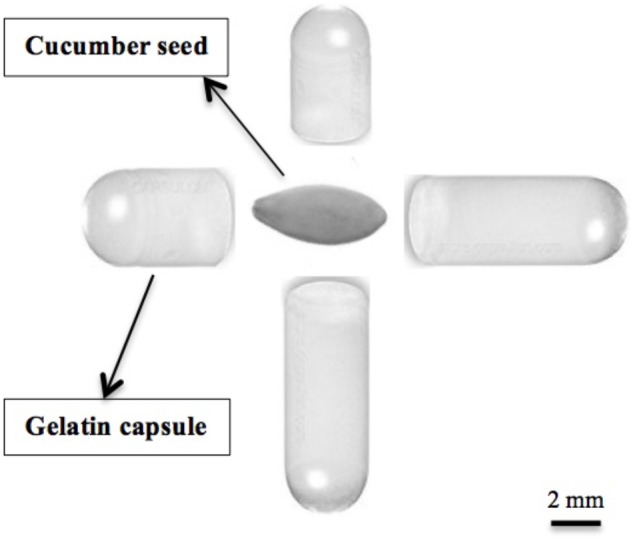
Two gelatin capsules (four halves) placed adjacent to the cucumber seed.

Six crops: cucumber “Vlaspik” (Seminis, Oxnard, CA, United States), arugula “Astro” (Sakata, Morgan Hill, CA, United States), broccoli “Centura” (Rogers, Boise, United States), tomato “Talladega” (Syngenta, Greensboro, NC, United States), pepper “Boynton Bell” (Harris Moran, Modesto, CA, United States), and field corn “Cornell D2901” (Cornell University, Ithaca, NY, United States) were grown in a controlled greenhouse maintained at 24°C/21°C temperature with 14/10 h photoperiod at New York State Agricultural Experiment Station in Geneva, NY, United States. Five gelatin capsule treatments (Capsule line, Pompano Beach, FL, United States) were evaluated using #3 hard-gel animal based gelatin capsules (Capsule line, Pompano Beach, FL, United States), referred throughout paper as a gelatin capsule or gelatin capsule treatments. One gelatin capsule contains 7.1 mg nitrogen that is equivalent to 40 mg protein (Gelatin Manufacturers Institute of America, 2012). Capsules were placed adjacent to each seed in a 10-cm square pot: control (no capsule), half capsule (0.5), whole capsule (1.0), two capsules (2.0), and three capsules (3.0). The placement of two capsules is illustrated in relation to a cucumber seed (**Figure 1**). Seeds were sown in 10 cm × 10 cm × 12 cm (1,200 cm^3^) plastic pots filled with Cornell Peat-Lite Mix A medium routinely used for seedlings or bedding plants. Peat-lite mix A is a combination of 1:2:2 (vol/vol) perlite: sphagnum peat moss: horticultural grade vermiculite with 4 kg of dolomitic limestone, 0.33 kg of nitrogen (N) and potassium (K_2_O), and 0.16 kg of phosphate (P_2_O_5_) per cubic meter of mix (Boodley and Sheldrake, 1972). Five replications of each treatment were placed in a random block design in the greenhouse. Plants were watered as needed every other day throughout the experiment. Plants were harvested 28 days after emergence and plant total leaf area and dry weight were measured. Total leaf area was measured using CI-202 Leaf Area Meter (CID Bio-Science, Camas, WA, United States).

### Experiment 2.1: Capsule Treatments and Growth Parameters of Cucumber Plants

Cucumber seeds “Vlaspik” (Seminis, Oxnard, CA, United States) were grown in a controlled greenhouse maintained at 24°C/21°C temperature with 14/10 h photoperiod at New York State Agricultural Experiment Station in Geneva, NY, United States. Four gelatin capsule treatments (Capsule line, Pompano Beach, FL, United States) were evaluated using #3 hard-gel gelatin capsules (Capsule line, Pompano Beach, FL, United States). Capsules were placed adjacent to each seed in a 10 cm square pot: control (no capsule), half capsule (0.5), whole capsule (1.0), two capsules (2.0). The placement of two capsules is illustrated in relation to a cucumber seed (**Figure 1**). Five replications of each treatment were placed in a random block design in the greenhouse, with 15 samples per replication. The plants were harvested 28 days after emergence and seven physiological parameters were measured (plant height, root length, leaf area, shoot fresh/dry weight, and root fresh/dry weight). Total leaf area was measured using CI-202 Leaf Area Meter (CID Bio-Science, Camas, WA, United States).

### Experiment 2.2: Capsule Treatments and Nitrogen Content of Cucumber Plant Tissue

Dry cucumber plant tissue was ground with a Wiley mill (Thomas Scientific Swedesboro, Swedesboro, NJ, United States) to a particle size of 2 mm. A 100 mg sample was sent to Cornell University Stable Isotope Laboratory (Ithaca, NY, United States) for elemental analysis to determine total nitrogen amount in plant tissue.

### Experiment 3: Analysis of Different Hydrolyzed Collagen Types Based on Molecular Weight Distribution

Ten gram samples of three different hydrolyzed collagen types: granulated 220 bloom gelatin, granulated gelatin capsule, and gelatin hydrolysate were submitted to Eastman Gelatine Co. (Peabody, MA, United States) for MWD analysis. MWD of hydrolyzed collagen was performed by high-performance liquid chromatography (HPLC) in the aqueous size exclusion mode (AEC). Gelatin samples were dissolved in the chromatographic eluent along with a phosphate buffer containing sodium dodecyl sulfate (SDS). Molecular weight fractions from experimental samples were separated on a TosoHaas TSK-Gel size exclusion column (TOSOH Co., Japan). The effluent was monitored with a UV detector set at 220 nm. Known molecular weight standards were run to prepare a calibration curve, which was constructed by plotting the log of these molecular weights versus the retention time. The MWDs of unknown gelatin samples were then determined from the linear portion of the calibration curve. Results were reported in terms of relative area percent of the five molecular weight regions (**Table 2**).

**Table 2:** Five molecular weight regions designated by the size exclusion elution profile and the corresponding molecular weight region of each fraction (Farrugia et al., 1998).

Fraction	Molecular weight region
High molecular weight fraction (HMW)	>250,000
Beta fraction	150,000–250,000
Alpha fraction	50,000–150,000
Sub alpha fraction	20,000–50,000
Low molecular weight fraction	4,000–20,000

### Experiment 4: Analysis of Different Hydrolyzed Collagens on Cucumber Plant Growth and Nitrogen Content

Cucumber seeds were planted in a controlled greenhouse maintained at 24°C/21°C temperature with 14/10 h photoperiod at New York State Agricultural Experiment Station in Geneva, NY, United States. Six treatments were compared; control, two gelatin capsules, hydrolyzed gelatin with bloom value of 220 (bloom 220), gelatin hydrolysate (Eastman Gelatine Co., Peabody, MA, United States), urea (Sigma-Aldrich, St. Louis, MO, United States), and an amino acid mixture that simulated the same composition as gelatin (Sigma-Aldrich, St. Louis, MO, United States) (**Table 1**). Bloom 220 gelatin was chosen as it has the same bloom value as the gelatin capsule. Gelatin hydrolysate was chosen for its small molecular size and its unique characteristic of lack of gelling capability. Two gelatin capsules were placed adjacent to each seed in a 10 cm × 10 cm × 12 cm pot (**Figure 1**). The hydrolyzed bloom 220 gelatin, gelatin hydrolysate, urea and amino acid mixture were weighed to the equivalent weight of two gelatin capsules (90 mg) and applied to the seed as powders in the pot. Each treatment had a total of 75 experimental units arranged in five replications of 15 experimental units per block, and the treatments were placed in a random block design on the greenhouse bench. The plants were harvested 28 days after emergence and leaf area and dry weight were measured. The plant nitrogen content was determined as previously described. The contribution of the gelatin treatments to nitrogen applied per plant was determined by capsule weight with 14% moisture content (Buice et al., 1995) and 17% of gelatin protein is nitrogen (Gelatin Manufacturers Institute of America, 2012).

### Experiment 5: Different Amino Acid Mixture Ratios and Cucumber Plant Growth

Proline and hydroxyproline in gelatin were investigated as the active components of the biostimulant response. Amino acid mixtures with different proportions of proline, hydroxyproline, and glycine were mixed to develop a coating powder (Wilson, 2015), which was then applied to cucumber seeds. The amino acid mixtures were designed to assess the effect of amino acids, proline, and hydroxyproline on the plant growth. They were selected because hydrolyzed collagen contains high concentrations of these two amino acids. Five different amino acid mixtures were used for seed coating: an amino acid mixture containing all the amino acids found in hydrolyzed collagen, all amino acids found in hydrolyzed collagen with proline replaced by glycine, all amino acids found in hydrolyzed collagen with hydroxyproline replaced by glycine, and all amino acids found in hydrolyzed collagen with proline and hydroxyproline replaced by glycine. A control contained no amino acid.

### Statistical Analysis

Data from experiments 1, 2, 4, and 5 were subjected to one-way analysis of variance (ANOVA). To detect the statistical significance of differences between means, the Tukey’s HSD test at a significance level of α = 0.05 was performed. Also for experiment 1, capsule treatments were grouped by crop species for analysis. Linear regression was used to study cucumber plant total nitrogen response to increasing nitrogen inputs from gelatin capsules (Experiment 2.2). For all experiments raw data were used for analyses. The statistical analysis was performed using the statistical program JMP Pro 11 (SAS Institute, Cary, NC, United States).

## Results

### Experiment 1: Capsule Treatments and Above Ground Growth of Selected Vegetable Crops and Field Corn

**FIGURE 2:**
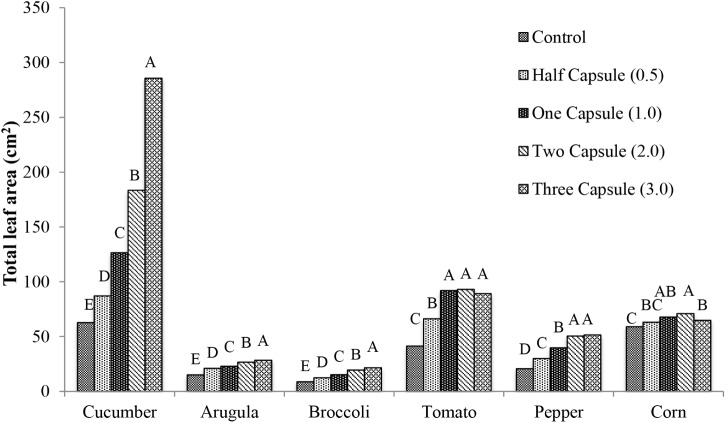
Mean total leaf area 28 days after emergence of six crops: cucumber, arugula, broccoli, tomato, pepper, and corn with five treatments: control, half capsule (0.5), whole capsule (1.0), two capsules (2.0), and three capsules (3.0). Data analyzed by ANOVA (α = 0.05) and Tukey’s HSD test was used to conduct mean separation comparison. Letters not associated with same letter are significantly different.

Enhanced plant growth was observed in the above ground portions of plants in treatments with gelatin capsules placed adjacent to seeds at the time of sowing. Results for total leaf area, and dry weight were reported as cm^2^ or g, respectively, and percent change with respect to the control. All of the crops exhibited significant increases in total leaf area with the gelatin capsule treatment. The magnitude of the effect of the capsule treatment on the total leaf area was specific to each crop (**Figure 2**). Cucumber had the most significant increases in total leaf area (*p* < 0.0001) with 25 cm^2^ (39% increase) with half capsule treatment, 64 cm^2^ (102% increase) with one capsule treatment, and 121 cm^2^ (193% increase) with two capsule treatment compared to the control. The maximum total leaf area in cucumber was achieved with the three capsule treatment with 223 cm^2^ (356% increase) compared to the control. Arugula and broccoli exhibited a similar increase in total leaf area with capsule treatments compared to cucumber. Tomato showed a significant increase in total leaf area with the addition of capsule treatments (*p* < 0.0001). For tomato there was a 50 cm^2^ (123% increase) with one capsule, and maximum increase of 52 cm^2^ (126%) with two capsule treatment, and a slight decrease in total leaf area with the three capsule treatment with 48 cm^2^ (116% increase). Pepper plants exhibited a similar trend as tomato. Corn did not respond as dramatically as the other crops and an increase of 12 cm^2^ (20%) was observed with the two capsule treatment.

**FIGURE 3:**
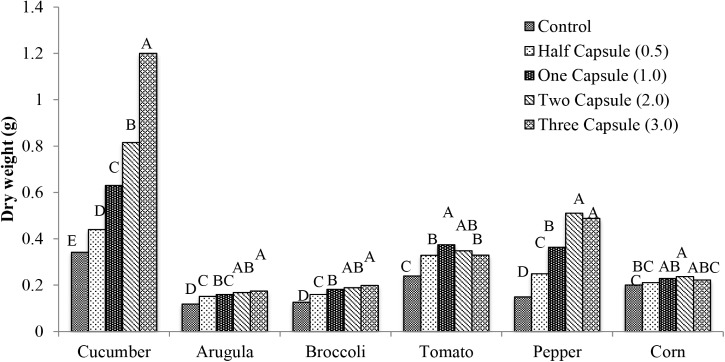
Mean dry weight 28 days after emergence of six crops: cucumber, arugula, broccoli, tomato, pepper, and with five treatments: control, half capsule (0.5), whole capsule (1.0), two capsules (2.0), and three capsules (3.0). Data analyzed by ANOVA (α = 0.05) and Tukey’s HSD test was used to conduct mean separation comparison. Letters not associated with same letter are significantly different.

Similar to total leaf area, all of the crops exhibited significant increases in dry weight with the capsule treatments (*p* < 0.005) (**Figure 3**) compared to the non-treated control. Cucumber had the most significant increases in dry weight (*p* < 0.0001) with 0.1 g (30% increase) with half capsule treatment, 0.29 g (84% increase) with one capsule treatment, and 0.47 g (138% increase) with the two capsule treatment compared to the control. The maximum dry weight in cucumber was achieved with the three capsule treatment with 0.86 g (251% increase) compared to the control. Arugula and broccoli exhibited similar increases in dry weight with capsule treatments compared to cucumber. Tomato had significant increase in dry weight with the addition of capsule treatments (*p* < 0.0001). There was 0.14 g (56%) maximum increase with one capsule, followed by a slight decrease in dry weight with the two and three capsule treatments with 0.11 g (45%) and 0.10 g (37%) increase, respectively. Pepper had a similar trend as tomato.

### Experiment 2.1: Capsule Treatments and Growth Parameters of Cucumber Plants

**FIGURE 4:**
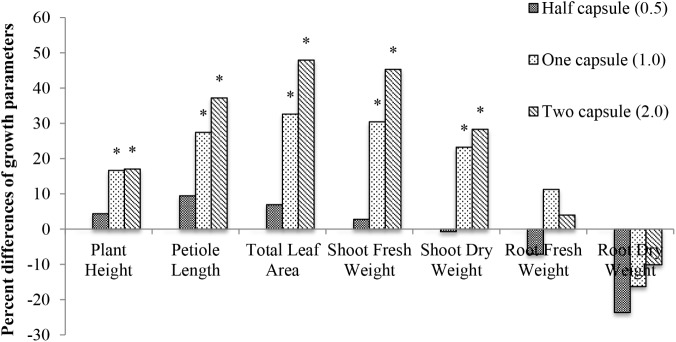
Percent difference of seven growth parameters (plant height, petiole length, total leaf area, shoot fresh weight, shoot dry weight, root fresh weight, and root dry weight) 28 days after emergence of cucumber with three treatments: half capsule (0.5), whole capsule (1.0), and two capsules (2.0) compared to control as a base line. ^∗^ Denotes significance (*p* < 0.05).

The effect of capsule treatments on cucumber plant was investigated by measuring seven different growth parameters (plant height, petiole length, total leaf area, shoot/root fresh weight, and shoot/root dry weight) 28 days after emergence. The half capsule treatment had the least effect on plant growth, and the two capsule treatments had the greatest effect on the overall cucumber plant growth compared to the non-treated control. An additive effect of the hard-gel capsule treatment was measured in plant height, petiole length, total leaf area, fresh and dry shoot weight. Data are presented as percent difference of the seven growth parameters compared to the control (**Figure 4**). The effects of the capsule treatments were not consistent for all plant parts. The above ground parts of the plant exhibited a significant increase (*p* < 0.05) with the addition of two capsule treatments. Petiole length increased 37%, total leaf area had a 48% increase, shoot fresh weight had a 45% increase (**Figure 4**). Below ground biomass did not reflect the same growth increase as measured for the above ground parts of the plant. There was no significant difference in growth for the below ground part of the cucumber plant.

### Experiment 2.2: Capsule Treatments and Nitrogen Content of Cucumber Plant Tissue

**FIGURE 5:**
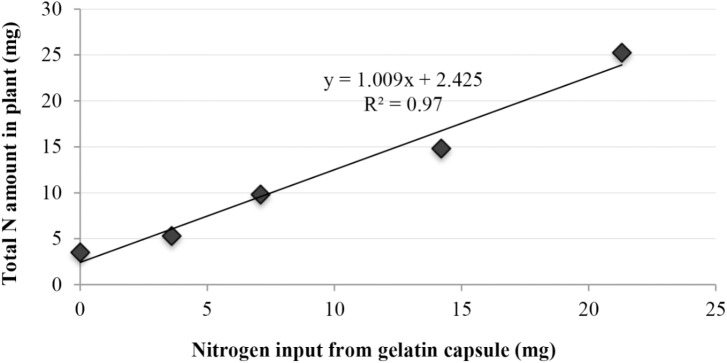
Linear regression with response of cucumber plant total nitrogen concentration (mg) to increasing nitrogen inputs (mg) from gelatin capsules.

Cucumber plants treated with gelatin capsules exhibited an increase in total nitrogen amount (mg) in plant tissue compared to the control (*p* < 0.0001) (**Figure 5**). The three capsule treatment exhibited the greatest percent increase in total nitrogen amount with 21.7 mg (617% increase), followed by treatment with two capsules 11.3 mg (321% increase), one capsule with 6.3 mg (178% increase), and the half capsule treatment with 1.8 mg (51%). Nitrogen amount recovered from plant tissues was significantly related to the nitrogen supplied by the gelatin treatments (*R*^2^ = 97.0%) (**Figure 5**).

### Experiment 3: Analysis of Different Hydrolyzed Collagen Types Based on Molecular Weight Distribution

**FIGURE 6:**
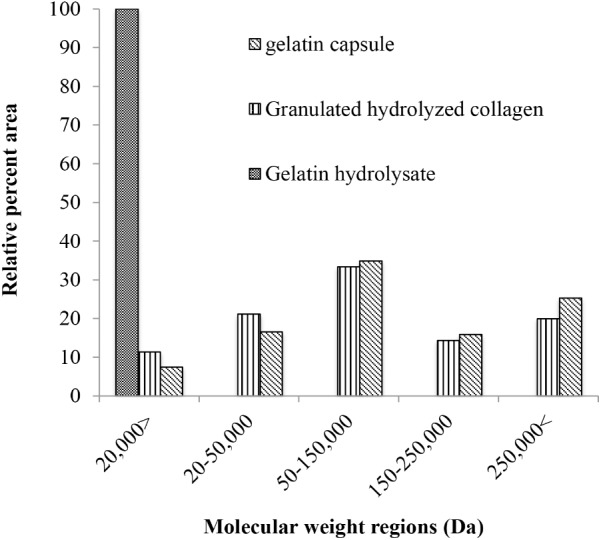
Molecular weight distribution of hydrolyzed collagen by type, reported in relative area percent of the five molecular weight regions the high molecular weight fraction, the beta fraction, the alpha fraction, the sub-alpha fraction, and the low molecular weight fraction of the size exclusion elution profile.

According to the size exclusion elution profile of the different hydrolyzed collagens, gelatin hydrolysate had the smallest molecular weight, and 100% of its fraction was in the low molecular weight fraction (**Figure 6**). The gelatin capsule had an elution profile similar to bloom 220 gelatin. The gelatin capsule and bloom 220 gelatin, termed granulated hydrolyzed collagen both had high molecular weight (HMW), molecular weight region (MWR) above 250,000 g mol^−1^, of 25 and 20%, respectively. The beta fraction, MWR between 250,000 and 150,000 g mol^−1^, was 16% for gelatin capsule, and 14% for granulated hydrolyzed collagen. The alpha fraction, MWR between 150,000 and 50,000 g mol^−1^, was 35% for gelatin capsule, and 33% for granulated hydrolyzed collagen. The sub alpha fraction, MWR between 50,000 and 20,000 g mol^−1^, was 16% for gelatin capsule, and 21.1% for the granulated hydrolyzed collagen. The low molecular weight fraction, MWR < 20,000 g mol^−1^, was 7.5% for gelatin capsule, and 11% for granulated hydrolyzed collagen. There were significant differences (*p* < 0.001) in the molecular distribution of the three hydrolyzed collagens. No significant difference was detected between gelatin capsule and granulated hydrolyzed collagen, while there were significant differences (*p* < 0.001) between gelatin capsule, granulated hydrolyzed collagen and gelatin hydrolysate.

### Experiment 4: Analysis of Different Hydrolyzed Collagens on Cucumber Plant Growth and Nitrogen Content

**FIGURE 7:**
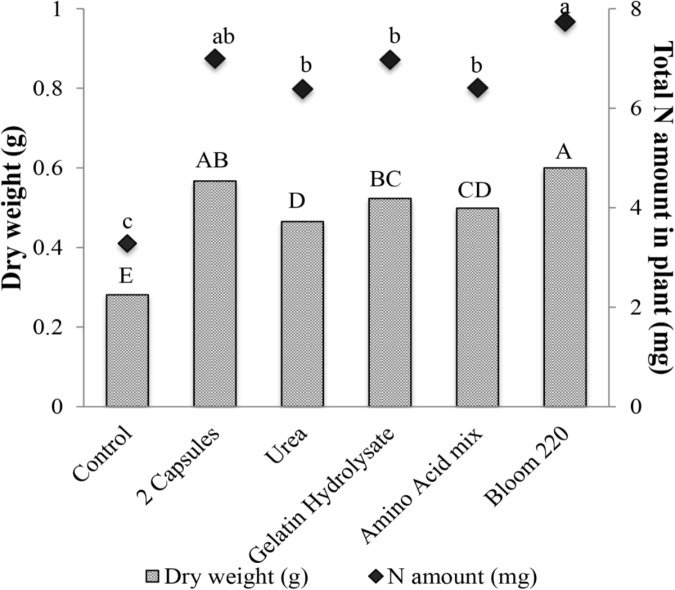
Mean dry weight (g) and total nitrogen amount in plant (mg), 28 days after emergence of cucumber with six treatments: control, two gelatin capsule (2 Capsules), urea, gelatin hydrolysate, amino acid mixture (Amino acid mix), and bloom 220 gelatin (Bloom 220). Data analyzed by ANOVA (α = 0.05) and Tukey’s HSD test was used to conduct mean separation. Letters (Capital letters for dry weight and small letters for N amount) not associated with same letter are significantly different.

The effects of four types of hydrolyzed collagen on plant growth were investigated by measuring total leaf area and dry weight. Only dry weight data are presented. The two capsules and bloom 220 treatments had the greatest dry weight compared to other treatments (**Figure 7**). The urea and amino acid mixture had similar dry weight, which was significantly greater than the non-treated control. The bloom 220 had the highest N amount per plant sample at 7.7 mg (140% increase), which was not significantly different than the two gelatin capsule treatment (**Figure 7**). There were no differences in the amount of nitrogen between the urea, gelatin hydrolysate and amino acid mixture treatments.

### Experiment 5: Different Amino Acid Mixture Ratios and Cucumber Plant Growth

**FIGURE 8:**
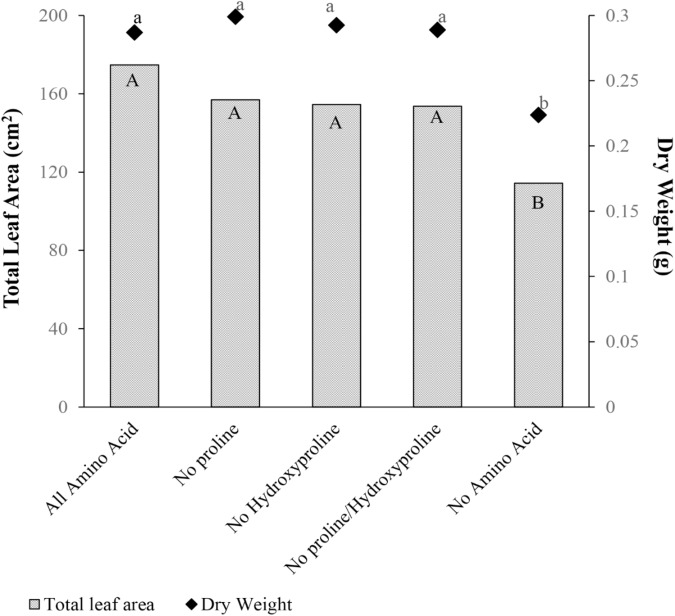
Mean total leaf area (cm^2^) and dry weight (g) 28 days after emergence of cucumber with five treatments: amino acid mixture containing all amino acids found in hydrolyzed collagen (All Amino Acid), all amino acids found in hydrolyzed collagen but proline replaced with glycine (No Proline), all amino acids found in hydrolyzed collagen but hydroxyproline replaced with glycine (No Hydroxyproline), and all amino acids found in hydrolyzed collagen but proline and hydroxyproline replaced with glycine (No Proline/Hydroxyproline), and Control (No Amino Acid). Data analyzed by ANOVA (α = 0.05) and Tukey’s HSD test was conducted for mean separation. Letters (Capital letters for total leaf area and small letters for dry weight) not associated with same letter are significantly different.

Proline and hydroxyproline were tested separately or in combination as a biostimulant. Total leaf area and dry weight were not different for the amino acid mixture containing proline, hydroxyproline or their combination, in comparison with the amino acid mixture without proline and/or hydroxyproline (**Figure 8**). Therefore, proline and hydroxyproline were not the primary amino acids responsible for the plant growth promotion from gelatin capsule treatments.

## Discussion

Six crops examined in this study (cucumber, arugula, broccoli, tomato, pepper, and corn) exhibited increased total leaf area and dry weight in response to gelatin capsule treatments (**Figures 2**, **3**). Two gelatin capsules placed adjacent to each seed increased dry weight of cucumber, pepper, broccoli, tomato, arugula, and field corn, by 138, 244, 50, 45, 41, and 18 percent, respectively, compared to the non-treated control (**Figure 3**). Therefore, all crops investigated exhibited enhanced plant growth; however, the growth enhancement, measured by the percent increase in dry weight, compared to the non-treated control differed by species. This may be attributed to the uptake mechanism of hydrolyzed collagen that is the main ingredient in the gelatin capsules. Hydrolyzed collagen, a polypeptide, is comprised of amino acids, and the amino acids can be used as a nitrogen source in plant nutrition (Schrieber and Gareis, 2007). Plants acquire nitrogen as nitrate and ammonium and as organic nitrogen in forms such as amino acids and protein from the soil (Nasholm et al., 2009; Tegeder and Rentsch, 2010).

Previous reports have shown that the uptake of amino acids and proteins can vary between plant species (Okamoto and Okada, 2004; Reeve et al., 2008). Okamoto and Okada (2004) investigated utilization of different forms of nitrogen (N) by four gramineous crops including sorghum (*Sorghum bicolor*), rice (*Oryza sativa*), maize (*Zea mays*), and pearl millet (*Pennisetum glaucum*). In their study, seedlings were grown without N, or with 500 mg N kg^−1^ applied to soil as ammonium nitrate, rice bran or a mixture of rice bran and straw. Shoot growth and N uptake in maize and pearl millet were correlated with the inorganic N (ammonium and nitrate) concentration in the soil, suggesting that these species depend on, or have a preference for, inorganic N. In contrast, shoot growth and N uptake patterns in sorghum and rice indicated that these two species could compensate for low inorganic N levels by taking up organic nitrogen (proteins) from the rice bran and straw mixtures. Analysis of N uptake in solution culture experiments confirmed that sorghum and rice roots were more able to absorb N in the form of protein than maize and pearl millet (Okamoto and Okada, 2004). Reeve et al. (2008) conducted a study on differential uptake of amino acid-N by domestic (*Fragaria fragaria*) and wild strawberry (*F. chiloensis* and *F. virginiana*). Exogenous glycine-N, NH_4_^+^-N, and NO_3_^−^-N was supplied to the three species of strawberry. The domestic strawberry only took up a small portion of N from the amino acids, while the wild species took up significantly more glycine-N and NH_4_^+^-N. These results show that utilization of organic N in the form of proteins and amino acids can be species specific.

In the study reported here, cucumber exhibited the largest growth response from gelatin capsule treatments than the other crops evaluated in the study. However, the gelatin capsule treatments only increased plant growth in the above ground biomass: plant height, petiole length, total leaf area, and shoot dry weight (**Figure 4**). Different doses of gelatin capsules had no significant effect on root growth compared to non-treated controls (**Figure 4**). The lack of the biostimulant effect on root growth may be related to the source of the protein in gelatin or the concentration and type that was available in the growing medium.

Research has shown that both the protein source and concentration applied can influence root growth and development. Lucini et al. (2018) measured root growth promotion using a plant-based biostimulant that contained lateral root promoting peptides in melon (*Cucumis melo* L.). In their experiments, five rates (0, 0.06, 0.12, 0.24, and 0.48 mL per plant) of a biopolymer biostimulant were applied as a substrate drench. Total root dry matter, maximum root length, and root surface area in biostimulant treated plants were the greatest at 0.24 mL plant^−1^, but decreased at 0.48 mL plant^−1^. Lonhienne et al. (2014) investigated externally supplied soluble proteins on root development of *Arabidopsis* and found that addition of low (1.6–5 μM) to intermediate (15–23 μM) concentrations of bovine serum albumen (BSA) protein increased root dry weight, root length and thickness, and root hair length. However, root growth was inhibited at the high concentration of BSA (45 μM).

A linear relationship between the nitrogen supplied by the gelatin capsules and the nitrogen in cucumber tissue was observed (**Figure 5**). Thus the increase in nitrogen in the plant tissue was partially attributed to increased nitrogen supplied by the gelatin capsule treatments. However, the results suggest that growth promotion induced by the gelatin capsules was not caused by the N fertility alone as the treatment with two gelatin capsules had greater dry weight than the amino acid mix with the same proportion and amount of amino acids as the gelatin treatment (**Figure 7**). To determine if the growth promotion by the gelatin capsules was caused by the fertilizer effect from the amino acids in the gelatin, urea was used as a treatment with the equivalent amount of N found in two gelatin capsules. Although urea has the potential to be phytotoxic, no adverse effect in these experiments measured as the growth response and nitrogen per plant were the same from the urea treatment and the amino acid mixture (**Figure 7**). Amirkhani et al. (2016) demonstrated that application of a plant protein to broccoli (*Brassica oleracea* L.) seeds as a seed coating resulted in an unexpected increase in N uptake. Application of soy flour (a plant-based biostimulant source of nitrogen) as a component of a seed-coating blend increased N uptake efficiency. Nitrogen, from the soy flour applied in the seed coatings ranged from 0.024 to 0.073 mg per seed, while the enhanced nitrogen per plant ranged from 1.7 to 8.5 mg. Nitrogen applied in the seed coating only accounted for 1–2% of the increased nitrogen in plants, indicating the soy flour acted as a biostimulant and not as a fertilizer.

The hydrolyzed collagens used in these experiments were grouped by molecular weight (**Table 2**), and their distribution differs between gelatin types (**Figure 6**). Gelatin hydrolysate had the smallest MWD with 100% of the elution in the low molecular weight fraction of <20 kDa (**Figure 6**). Gelatin capsules used in these experiments had an elution profile similar to granulated hydrolyzed collagen, which suggests that they are structurally similar. This speculation is supported by the plant dry weight results from the different hydrolyzed collagens that were evaluated. There were no significant differences in dry weight of cucumber between application of two gelatin capsules, and granulated hydrolyzed collagen (Bloom 220), while the dry weight for gelatin hydrolysate was significantly less than the Bloom 220 treatment (**Figure 7**). One explanation for the difference in plant dry weight between the treatments may be the water solubility of the different hydrolyzed collagens. Both gelatin hydrolysate and the amino acid mix have a high water solubility. As the plants were watered in the greenhouse during the experiments, the treatments that were applied in dry powder form may have dissolved in the water and leached from the containers. The lower solubility collagens may have remained in the container for a longer period of time and thus available for plant growth resulting in a higher plant biomass. Additional research is warranted to investigate water solubility and duration of availability of the different treatments for plant uptake.

The underlying mechanism of plant growth enhancement in response to gelatin capsules remains unclear. Gelatin has a unique composition of amino acids, and is rich in proline and hydroxyproline (**Table 1**). Vaughan and Cusens (1973) showed that application of hydroxyproline enhanced root growth of peas. They proposed that externally supplied hydroxyproline enhanced root extension growth by affecting cell wall synthesis (Vaughan, 1973; Vaughan and Cusens, 1973). However, treatments with no proline and/or hydroxyproline were not significantly different than treatments with all amino acids (**Figure 8**). Therefore, proline and hydroxyproline were not the primary amino acids responsible for the plant growth promotion from gelatin capsule treatments.

Protein hydrolysates have been shown to stimulate carbon and nitrogen metabolism and increase nitrogen assimilation in plants (Schiavon et al., 2008; Ertani et al., 2009). Nitrate reductase, NAD-dependent glutamate dehydrogenase, and malate dehydrogenase increased in maize following application of animal epithelial hydrolysate (Maini, 2006). Alfalfa protein hydrolysate applied to hydroponically grown maize increased the activity of malate dehydrogenase, isocitrate dehydrogenase, and citrate synthase; as well as nitrogen metabolism enzymes, nitrate reductase, nitrite reductase, glutamine synthetase (GS), glutamine oxoglutarate aminotransferase (GOGAT), and aspartate aminotransferase (Schiavon et al., 2008). Ertani et al. (2009) reported that both alfalfa protein hydrolysate and animal connective tissue hydrolysate stimulated plant growth, and also increased nitrate conversion into organic nitrogen by inducing nitrate reductase and GS activities. These results suggest that protein hydrolysates may enhance plant growth by up regulating nitrate assimilation metabolism (Ertani et al., 2009; Calvo et al., 2014; Colla et al., 2017; Rouphael et al., 2017). Research in our lab reported that gelatin capsule treatment increased expression of amino acid and nitrogen transporter genes that may be responsible for root nitrogen uptake enhancement. Wilson et al. (2015) compared non-treated cucumber seeds and gelatin treated cucumber seeds using RNA-seq data. Amino acid permease 3 (AAP3), an amino acid transporter, was upregulated with the gelatin capsule treatment. The AAP3 gene is preferentially expressed in the phloem and has been associated with long distance transport of basic amino acids such as arginine, histidine, and lysine. The increased expression of amino acid transporters AAP3 and amino acid permease 6 (AAP6) in the plants treated with gelatin capsules suggests that amino acid transport in the plants was positively enhanced by the gelatin treatment. Our results provide evidence that proteins of the hydrolyzed collagen present in gelatin capsules provided a sustained source of N and acted as a biostimulant. Further research is needed to fully elucidate the mechanisms involved with the effect of gelatin on plant nutrition and growth.

## Author Contributions

HW performed the experiments and wrote many parts of the article. MA involved in data analysis, results interpretation, and writing the article. AT defined the scientific hypothesis and set up the experiments protocol and contributed to refinement and development of this paper.

## Conflict of Interest Statement

The authors declare that the research was conducted in the absence of any commercial or financial relationships that could be construed as a potential conflict of interest.
